# A rare case of Takotsubo syndrome with ventricular septal rupture: Case report

**DOI:** 10.1097/MD.0000000000031674

**Published:** 2022-11-25

**Authors:** Hamada Alsheikh, Nour Shaheen, Wageih Saber, Mostafa Meshref, Yara Amro, Ahmed Shaheen, Mahmoud Galal Ahmed, Sarya Swed

**Affiliations:** a Al-Azhar University, Cairo, Egypt; b Alexandria University, Alexandria Faculty of Medicine, Alexandria, Egypt; c Al-Azhar University, Cairo, Egypt; d Al-Azhar University, Cairo, Egypt; e Ministry of Health, Egypt; f Alexandria University, Alexandria Faculty of Medicine, Alexandria, Egypt; g Neurology Department, Faculty of Medicine, Al-Azhar University, Cairo, Egypt; h Faculty of Medicine, Aleppo University, Aleppo, Syria.

## Abstract

Takotsubo cardiomyopathy (TCM) mimics acute coronary syndrome and is characterized by reversible left ventricular (LV) apical ballooning in the absence of angiographically substantial coronary artery stenosis.

**Patient concerns:**

A 31-year-old man with acute dejection, physical stress, and psychological strain from the dread of losing his work arrived at the emergency department with chest pain, and discomfort that had lasted 3 hours.

**Diagnosis:**

Once the coronary angiography revealed normal epicardial coronaries, the case was retroactively diagnosed, and the levels of cardiac enzymes were increased.

**Interventions:**

The amount of necrotic tissue was so little that the surgeon could only verbally convey it. It is completely closed with the help of a Dacron sheet. The patient received surgical closure of the VSR a few days after having a surgical consultation.

**Outcomes:**

No postoperative echocardiogram was required, and the patient was sent home in great general condition.

**Conclusion:**

The presence of TCM with a ruptured LV wall was extremely rare because our patient had neither clinical risk indicators nor a family history of coronary artery disease. As a Takotsubo syndrome severe complication, we underline the significance of identifying, diagnosing, and treating it.

## 1. Introduction

Takotsubo syndrome is a condition that originated in the 1990s; it is an acute form of heart failure that presents with symptoms similar to a heart attack. The condition is also known as “broken heart syndrome, “acute stress-induced cardiomyopathy, or “apical ballooning.”^[1]^ Symptoms of the disorder resemble acute myocardial infarction in that it is characterized by temporary left ventricular (LV) dysfunction, abnormal wall motion, and elevated cardiac biomarkers.^[2]^ The diagnostic criteria classify Takotsubo cardiomyopathy (TCM) as a syndrome of unknown etiology, characterized by acute dilation of the LV apex. Chest pain and dyspnea are the most common symptoms.^[3]^ Most TCM patients are postmenopausal women presenting with the acute coronary syndrome.^[4]^ TCM can be associated with stressful emotional or physical events in nearly two-thirds of patients.^[6]^ However, in this case, report, we have a rare condition of TCM with a transient dysfunction of the LV apex accompanied by emotional and physical stress, after which it is resolved. Takotsubo syndrome was classified as acquired cardiomyopathy by the American Heart Association in 200.^[5]^

TCM is diagnosed in 0.02% of all nationwide hospitalizations and has an inpatient mortality rate of 4.5%.^[6]^ According to a recent TCM registry, the rate of major adverse cardiac and cerebrovascular events during long-term follow-up was 9.9%, and the rate of death was 5.6%.^[7]^ Despite TCM’s self-limiting nature, it may present rare complications like systemic embolism, ventricular arrhythmias, cardiogenic shock, and LV rupture resulting in cardiac arrest.^[8]^ We describe a very unusual case of a patient with Takotsubo syndrome and an acute ventricular septal defect (VSD) simultaneously.

## 2. Case presentation

**Figure 1.**
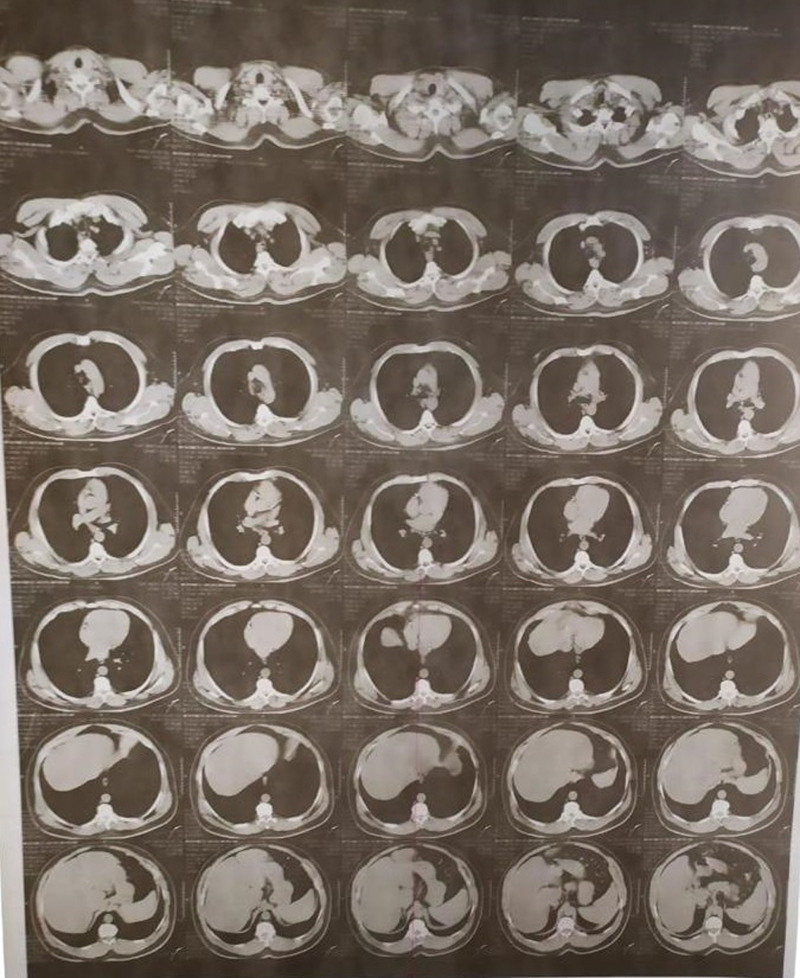
Chest CT of the patient with Takotsubo syndrome.

**Figure 2.**
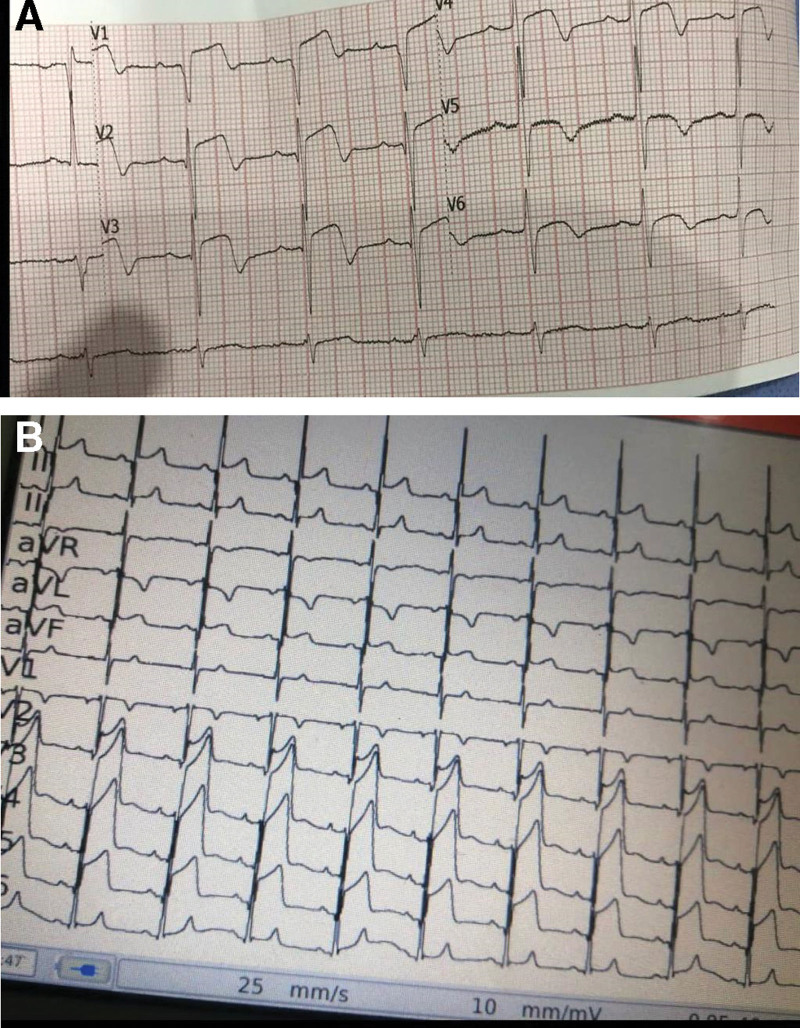
Electrocardiogram (ECG) of a patient with Takotsubo cardiomyopathy (TCM) 1st and 2nd day of presentation.

A 31-year-old man arrived at the emergency department with 3-hour-long chest discomfort caused by severe despair and physical and psychological stress from the fear of losing his job. The patient had visited the emergency room the previous day with complaints of chest discomfort. The patient had no family history of heart illness and no risk factors for coronary artery disease. He does not smoke and has never been a drug addict or participated in substance abuse. There is no audible murmur because of the following factors: it is a large VSR (large VSD frequently present without a murmur); it is an apical VSR (unusual site) in the trabecular parts of the IVS; impaired LV function by MI; this results in no or very little pressure gradient between the ventricles; and it may have been overlooked by the doctor who examined the patient. A negative swab and chest computed tomography had ruled out COVID-19 infection (Fig. 1). The electrocardiograms (ECG) lead a VL and lead I showed widespread and noticeable ST elevation (Fig. 2a and b). The ECG showed ST elevation in all pericardial leads with reciprocal changes in the inferior leads suggesting anterior ST segment elevation myocardial infarction (STEMI).

**Figure 3.**
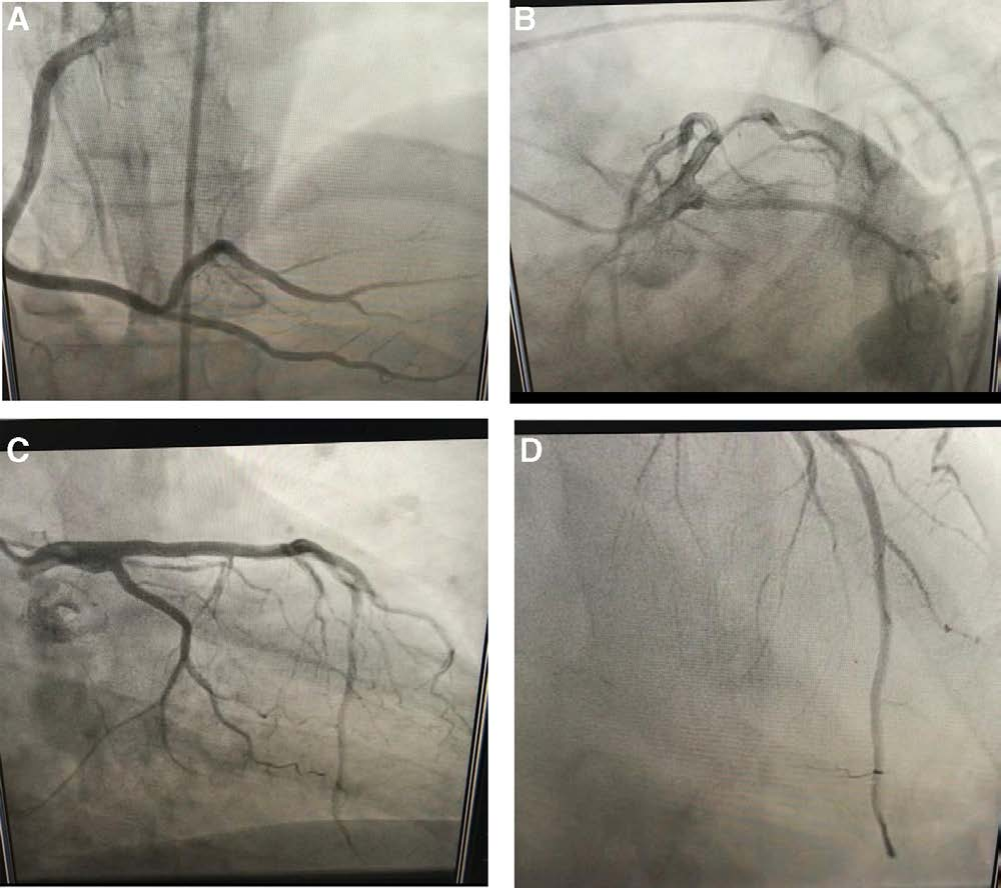
(a, b, c, d) Coronary angiography of the patient with Takotsubo syndrome.

**Figure 4.**
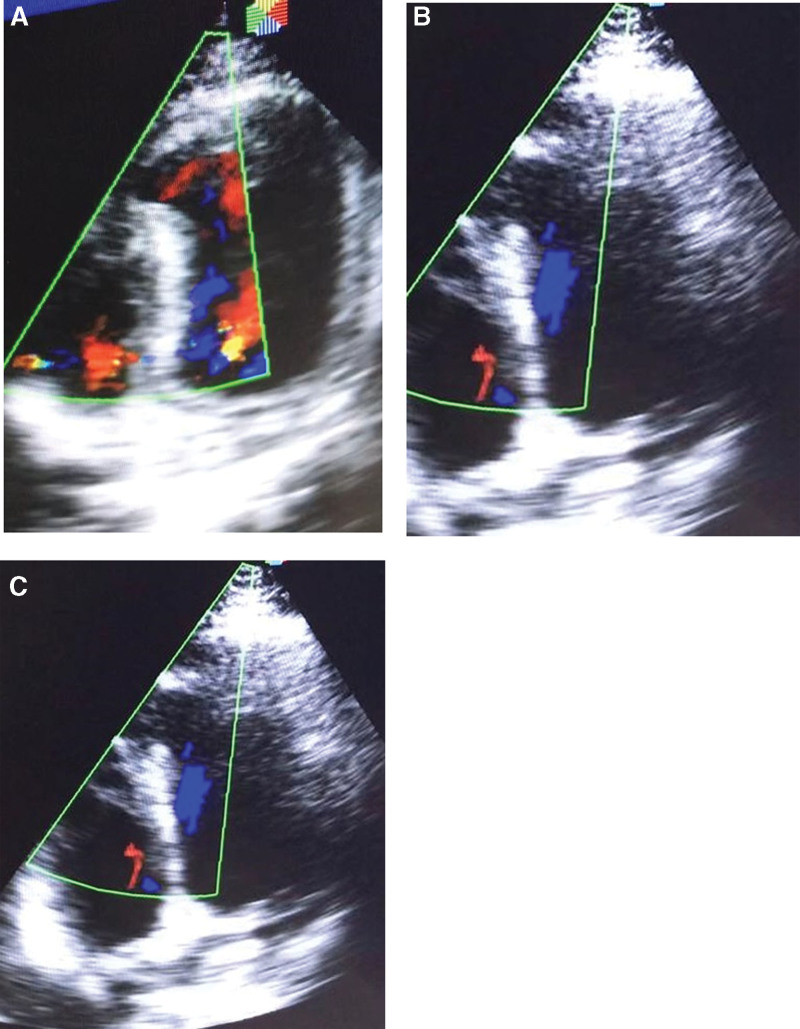
(a, b, c) Echocardiography of a patient with Takotsubo syndrome.

The patient was taken to Cath-Lab. For primary percutaneous coronary intervention. The case was diagnosed retrogradely when the coronary angiography showed normal epicardial coronaries. Cardiac enzymes have increased levels (Table 1). The existence of arterial thrombosis, especially pulmonary embolism, was ruled out by the D-dimer test’s low positivity (negative, by the local lab reference). COVID-19 infection was ruled out by radiographic and laboratory testing. The patient had an anterior STEMI or acute coronary syndrome. Following the American and European recommendations for revascularization of the STEMI using the main percutaneous coronary intervention method, the patient was brought immediately to the Cath-lab.^[9]^ Unexpectedly, the coronary angiography findings were normal (Fig. 3a–d). Echocardiography results revealed a kinetic and inflated apex, a dyskinetic inferior septum, hyperkinetic basal LV segments, and a massive VSR in the apical-inferior septum with L-R shunt that mostly occurs during diastole, and other abnormalities. The left ventricle was not dilated, and there was no evidence of elevated pulmonary artery pressure (which was not a congenital VSD) (Fig. 4a and b).

**Table 1:** Cardiac biomarkers of the patient with Takotsubo syndrome.

	The value	Normal range
Nt-Pro BNP	927 pg/mL	<125 pg/mL
Troponine	>40,000 ng/L	0< >0.04 ng/mL
D-Dimer	232 ng/mL	<0.50 ng/mL

Unfortunately, the surgery information is not obtainable. The defect in the apical-inferior septum was 2.6 × 1.9 cm. However, the surgeon could only verbally describe how little necrotic tissue was present. A Dacron sheet effectively closes it, leaving no residue. All labs, including the D-Dimer test, have normal ranges apart from elevated cardiac biomarkers. Hemodynamically stable (BP was 110/60 mm Hg, Pulse was ~80 bpm and regular, RR was ~20 cpm and no fever), the patient managed the situation with little to no symptoms. After receiving a surgical consultation, the patient had surgical closure of the VSR a few days later. The patient was sent home in excellent overall health with no need for postoperative echocardiography. The patient received follow-up care and a psychiatric evaluation and is enrolled in a rehabilitation program.

## 3. Discussion

Several studies have described LV dysfunction as reversible without coronary artery stenosis, with symptoms similar to acute myocardial infarction.^[10]^

In our case, the left ventricle dimensions are not dilated on the echocardiographic study and show the absence of apical ballooning with no evidence of LV mural thrombus as the echocardiographic findings suggest, that it’s not a congenital VSD (Time of presentation is very delayed. No signs of LV remolding and PHTN. This is an unusual site for congenital VSD). Furthermore, very high troponin levels and early enzyme peaking are typical observations in MINOCA (MI in non-obstructive CAD), which is caused by the washout of the metabolites into the serum in the absence of restricted coronary flow.

Stress-induced TCM causes an acute ventricular apical ballooning of unknown cause.^[11]^ The pathophysiology of myocardial stunning is unknown, but catecholamines are a likely cause. Chest pain and dyspnea are typical symptoms.^[12]^ The ECG can show transient ST elevation, and a small rise in cardiac troponin T is invariable. Patients with LV hypokinesis or akinesis have preserved basal systolic function without obstructive coronary lesions.^[13]^ Most patients recover quickly, and the prognosis is generally good, like in our presented case.^[11]^ TCM usually is a self-limited disease with rapid resolution of symptoms and LV dysfunction. Patients with TCM typically have a good prognosis; 96% recover from their symptoms almost completely, and the hospital mortality varies between 1% and 2%.^[14,15]^

Echocardiography can be crucial in diagnosing and following Takotsubo syndrome, as demonstrated in our case, as it can detect the classic wall motion pattern and other atypical variants (16). Besides speckle-tracking and contrast echocardiography, stress echocardiography can also provide further insight into the overall pathophysiology and mechanisms of dysfunction in this syndrome. Our previously healthy patient presented with acute myocardial infarction, demonstrating the classic features of Takotsubo syndrome: demographic of no risk factors for coronary artery disease, no family history of cardiac disease. He is a nonsmoker, and neither is he addicted to drugs nor has he ever abused substances.

Despite the initial diagnosis of a subacute MI, his case was not consistent with an infarction for several reasons: most importantly, his coronaries were completely normal without any stenosis or luminal irregularities, and on follow-up, EKGs showed no Q-waves, and post-recovery echocardiograms showed normal wall thickness without evidence of residual wall motion abnormalities. The etiology of his acute VSD is likely to be secondary to ventricular septal perforation caused by Takotsubo syndrome.

The development of VSD after Takotsubo syndrome is extremely rare and not as well understood, but its insight and pathology suggest a similarly poor prognosis without intervention.

## 4. Conclusion

Due to our patient had no clinical risk factors and no family history of coronary artery disease, the existence of TCM with LV wall rupture was highly unusual. In this instance, despite the widespread belief that it is a mild illness, we emphasize the significance of detecting, diagnosing, and treating it as a complication of Takotsubo syndrome.

## Author contributions

All authors have contributed in writing and revising the manuscript.

**Writing – original draft:** Hamada Alsheikh, Nour Shaheen, Sarya Swed.

**Writing – review & editing:** Wageih Saber, Mostafa Meshref, Yara Amro, Ahmed Shaheen, Mahmoud Galal Ahmed.
